# Genome Sequence of Type Strain *Bacillus decisifrondis* E5HC-32^T^ (DSM 11725^T^), Isolated from Soil Underlying the Decaying Leaf Litter of a Slash Pine Forest

**DOI:** 10.1128/genomeA.01249-15

**Published:** 2015-10-22

**Authors:** Guo-hong Liu, Bo Liu, Jie-ping Wang, Qian-Qian Chen, Jian-Mei Che, Xue-fang Zheng, Ci-bin Ge

**Affiliations:** Agricultural Bio-Resources Research Institute, Fujian Academy of Agricultural Sciences, Fuzhou, Fujian, China

## Abstract

*Bacillus decisifrondis* E5HC-32^T^ (DSM 11725^T^) is a Gram-positive, subterminal spherical spore-forming, strictly aerobic bacterium. Here, we report the 5,613,728-bp genome sequence of *B. decisifrondis* E5HC-32^T^, which is the first genome information of this species and will provide useful information for genomic taxonomy and phylogenomics of *Bacillus*-like bacteria.

## GENOME ANNOUNCEMENT

The type strain E5HC-32^T^ was isolated from soil in Australia and was named as *Bacillus decisifrondis* sp. nov. (1). Its phylogenetically close neighbors are *Lysinibacillus xylanilyticus* (2) and *Lysinibacillus macroides* (3) according to their 16S rRNA gene sequence similarity. The best growth of *B. decisifrondis* E5HC-32^T^ was achieved at 30°C and pH 8.4 (1). Notably, there is no other information about *B. decisifrondis* outside its taxonomical description information so far. Given no available genomic information of *B. decisifrondis*, its type strain E5HC-32^T^ was selected as one of the research objects in our “genome sequencing project for genomic taxonomy and phylogenomics of *Bacillus*-like bacteria.” Here, we present the high-quality draft genome sequence of *B. decisifrondis* E5HC-32^T^ (DSM 11725^T^).

The genome sequencing of *B. decisifrondis* E5HC-32^T^ (DSM 11725^T^) was performed via the Illumina Hiseq 2500 system. Two DNA libraries with insert sizes of 500 bp were constructed and sequenced. After filtering of the 0.67-Gb raw data, 0.66-Gb of clean data was obtained, providing approximately 100-fold coverage. The reads were assembled via SOAPdenovo software version 2 (4), using a key parameter K setting at 77. Through the data assembly, 78 scaffolds with a total length of 5,613,728 bp were obtained, and the contig *N*_50_ was 85767 bp. The average length of the scaffolds was 71,970 bp, and the longest and shortest scaffolds were 469,187  and 591 bp, respectively. A total of 89.95% clean reads were aligned back to the genome, which covered 99.01% f the sequence,

The annotation of the genome was performed using the NCBI Prokaryotic Genomes Automatic Annotation Pipeline (PGAAP) (http://www.ncbi.nlm.nih.gov/genomes/static/Pipeline.html) utilizing GeneMark, Glimmer, and tRNAscan-SE tools (5). A total of 5,439 genes were predicted, including 5,057 coding sequences (CDS), 294 pseudogenes, 82 tRNAs, and 6 rRNA genes. There were 3,510 and 2,375 genes assigned to the COG and KEGG databases, respectively. The average DNA G+C content was 36.60%, agreeing with the value 41 ± 1 mol% as determined by the thermal denaturation method (1).

### Nucleotide sequence accession numbers.

This whole-genome shotgun project has been deposited at DDBJ/EMBL/GenBank under the accession no. LIYL00000000. The version described in this paper is version LIYL00000000.1.
